# Toward Accurate Ab Initio Ground-State Potential Energy and Electric Dipole Moment Functions of Carbon Monoxide

**DOI:** 10.1021/acs.jctc.4c01082

**Published:** 2024-10-01

**Authors:** Jacek Koput

**Affiliations:** Department of Chemistry, Adam Mickiewicz University, 61-614 Poznań, Poland

## Abstract

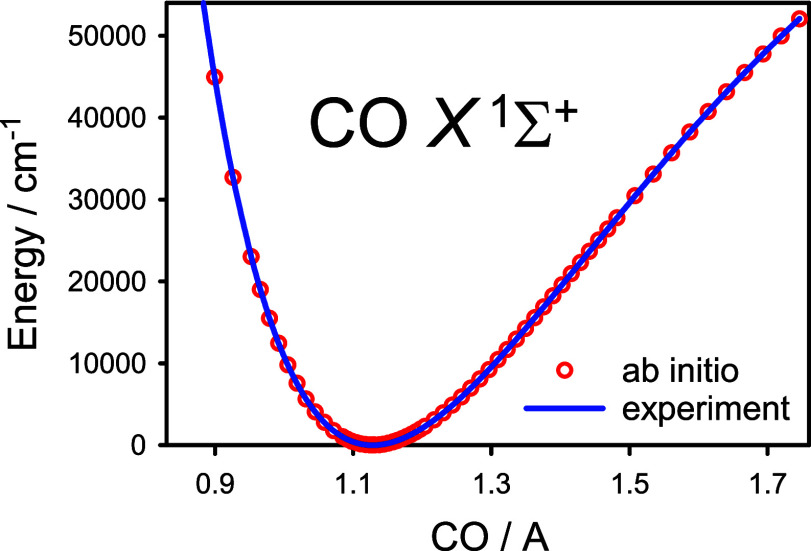

Accurate potential energy and electric dipole moment functions of the CO molecule in its ground electronic state X^1^Σ^+^ have been obtained using the single-reference coupled-cluster approach, up to the CCSDTQP level of approximation, in conjunction with the augmented core–valence correlation-consistent basis sets, aug-cc-pCV*n*Z, up to octuple-zeta quality. The scalar relativistic, adiabatic, and nonadiabatic effects were discussed. The ab initio predicted functions were compared with their experimentally derived counterparts.

## Introduction

Carbon monoxide, CO, is a simple heteronuclear closed-shell molecule. Because it consists of only two nuclei and ten valence electrons, the CO molecule is an attractive benchmark molecule for high-level ab initio calculations. On the other hand, the triple C≡O bond is the strongest bond (in terms of the dissociation energy) currently known among stable diatomic molecules, with the dissociation energy *D*_0_ being 1072 kJ/mol.^1^ Therefore, the CO molecule is also a challenge for an accurate theoretical description along with the well-known isoelectronic dinitrogen molecule.

On the experimental side, spectroscopic properties of the CO molecule in its ground electronic state X^1^Σ^+^ received increased attention in recent years.^2−8^ As carbon monoxide is the second most abundant compound in the universe, accurate spectroscopic parameters of the CO molecule play a pivotal role in the remote sensing of terrestrial and stellar atmospheres. However, even a diatomic molecule is a challenge for the determination of the molecular parameters, included in the molecular Hamiltonian, from high-resolution spectral data. As discussed in detail by Watson,^9^ the molecular parameters can be unambiguously determined only within the framework of the Born–Oppenheimer (BO) approximation. Beyond this approximation, there is indeterminacy in the treatment of BO breakdown effects. In an analysis of experimental spectral data, one of the possible solutions to this issue is morphing molecular parameters estimated in theoretical calculations. Such a semiempirical approach was applied recently by Špirko^8^ to determine the electric dipole moment function of CO. Clearly, the reliability of the morphing procedure depends on the quality of theoretical predictions.

This study aims to provide accurate state-of-the-art potential energy and electric dipole moment functions for the ground electronic state of CO. The molecular functions describing the adiabatic and nonadiabatic effects of CO were also determined. All of these functions were compared to their experimentally derived counterparts.

## Results and Discussion

The vibration–rotation energies and wave functions of CO were determined using the effective Hamiltonian *Ĥ*_eff_(*r*)^10−13^:
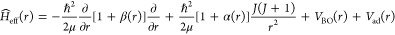
1where *r* is the internuclear distance, μ is the nuclear reduced mass, *V*_BO_(*r*) is the BO potential energy function, and *V*_ad_(*r*) is the diagonal (adiabatic) energy correction. The functions α(*r*) and β(*r*) represent nonadiabatic second-order perturbational corrections to the potential and kinetic energy parts of the Hamiltonian, respectively. Further nonadiabatic corrections to the potential energy part are neglected. The corresponding Schrödinger equation is solved for each value of the rotational quantum number *J*, the solutions being labeled with the vibrational quantum number *v*.

The potential energy function *V*_BO_(*r*) of CO was first determined using the single-reference coupled-cluster method,^14−18^ including single and double excitations and a perturbational correction due to connected triple excitations, CCSD(T). The augmented correlation-consistent core–valence basis sets up to octuple-zeta quality, aug-cc-pCV*n*Z (*n* = 5–8), were employed.^19−22^ All 14 electrons of the CO molecule were included in the correlation treatment. The calculations were performed using the MOLPRO^23^ and DALTON^24^ programs. The total electronic energies of CO were calculated at 66 internuclear distances *r* ranging from 0.8 to 1.8 Å, with the energy extending up to about 79,000 and 52,000 cm^–1^ (above a minimum) at the lower and upper *r* limits, respectively. The *r* region chosen covers about 60% of the well depth of CO. As shown by Coxon and Hajigeorgiou,^2^ such a potential energy function of CO supports the vibration energy levels up to *v* = 27. The equilibrium internuclear distance *r*_e_ was determined by fitting the predicted total electronic energies, in the vicinity of the minimum, with a polynomial expansion. The vibration–rotation energy levels of the main isotopologue ^12^C^16^O were then determined for the rotational quantum number *J* ranging from 0 to 3. The Numerov–Cooley method^25^ was applied to obtain eigenvalues and eigenfunctions of the effective Hamiltonian *Ĥ*_eff_. For a given vibrational state, the effective rotational constant *B*_v_ was determined by fitting the predicted rotational energies with a power series in *J*(*J* + 1). The electric dipole moment of CO was determined by using the finite field approach. The total electronic energy of the CO molecule in a static homogeneous electric field was calculated and used to obtain the dipole moment value with the five-point central difference formula. The predicted molecular parameters of CO are given in Table 1. The predicted values tend clearly to converge with an enlargement of the one-particle basis set. In particular, the total electronic energy of CO at the minimum is converged to better than 0.9 m*E*_h_. Concerning the basis set size, the best predicted equilibrium distance *r*_e_ is estimated to be accurate to better than ±0.0001 Å. The analogous error bars for the vibrational fundamental wavenumber ν, the effective ground-state rotational constant *B*_0_, and the electric dipole moment μ (at *r* = 1.1282 Å, close to *r*_e_) are estimated to be ±0.3 cm^–1^, ±0.0003 cm^–1^, and ±0.0004 D, respectively.

**Table 1:** Molecular Parameters for the X^1^Σ^+^ State of ^12^C^16^O Determined at the CCSD(T)/aug-cc-pCV*n*Z Level of Theory

	*n* = 5	*n* = 6	*n* = 7	*n* = 8
*r*_e_^a^ (Å)	1.12836	1.12804	1.12791	1.12784
*E* + 113^b^ (hartree)	–0.316416	–0.320303	–0.322126	–0.323070
ν^c^ (cm^–1^)	2147.80	2149.26	2150.06	2150.39
*B*_0_^d^ (cm^–1^)	1.92300	1.92408	1.92454	1.92479
μ^e^ (D)	–0.11901	–0.11791	–0.11732	–0.11694

a The equilibrium internuclear distance.

b The total energy at a minimum.

c The vibrational fundamental wavenumber.

d The ground-state effective rotational constant.

e The electric dipole moment at *r* = 1.1282 Å.

**Figure 1:**
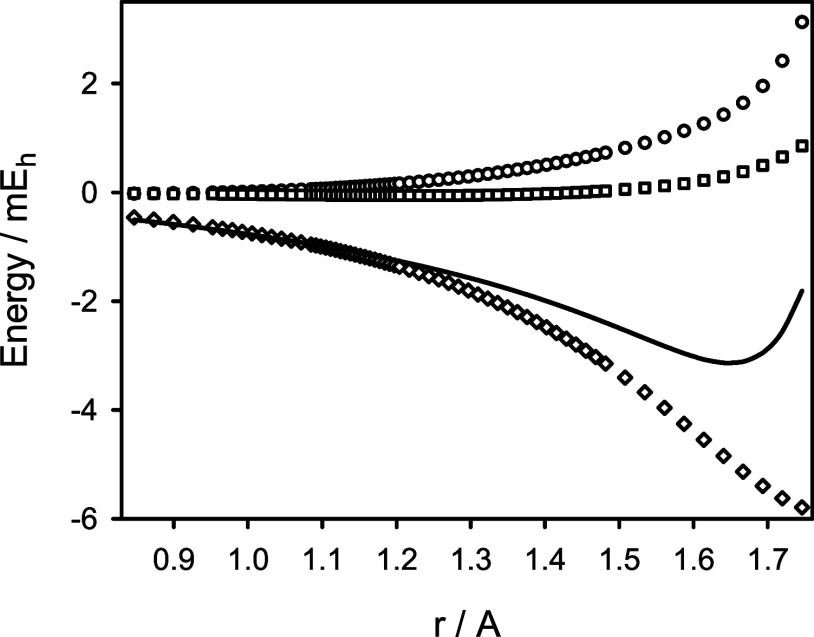
Predicted higher-order electron correlation corrections to the total electronic energy for the X^1^Σ^+^ state of ^12^C^16^O as functions of the internuclear distance *r* (see text): *T* – (*T*) (circles), *Q* (diamonds), *P* (squares), and the sum [*T* – (*T*)] + *Q* + *P* (solid line).

Changes in the potential energy function *V*_BO_(*r*) due to electron correlation beyond the CCSD(T) level of approximation were estimated in the subsequent calculations up to the CCSDTQP level. First, a difference between the iterative and perturbational treatments of connected triple excitations was considered. At each grid point mentioned above, the difference *E*[CCSDT/aug-cc-pCV5Z] – *E*[CCSD(T)/aug-cc-pCV5Z] was calculated, where *E*[···] denotes the total electronic energy of CO at a given level of theory. All of the electrons of CO were included in the correlation treatment, and this energy correction was referred further to as “*T* – (*T*)”. The second energy correction accounted for the correlation effect of connected quadruple excitations. It was calculated as the difference *E*[CCSDTQ/aug-cc-pVQZ] – *E*[CCSDT/aug-cc-pVQZ], with only valence electrons of CO being correlated, and is referred to as “*Q*”. Next, the correlation effect of connected pentuple excitations was investigated. The difference *E*[CCSDTQP/aug-cc-pVDZ] – *E*[CCSDTQ/aug-cc-pVDZ] was calculated, and it is referred to as “*P*”. The calculations were performed using the CFOUR^26^ and MRCC^27^ programs. A comparison of these three higher-order electron correlation corrections as functions of the internuclear distance *r* is illustrated in Figure 1. In the vicinity of the equilibrium configuration of CO, the *T* – (*T*), *Q*, and *P* energy corrections amount to about 0.09, −1.09, and −0.06 m*E*_h_, respectively. The *Q* energy correction definitely dominates along the *r* region under consideration. Because the *P* energy corrections were found to be at least an order of magnitude smaller, energy corrections due to connected hextuple excitations were not considered. Near the upper *r* limit (*r* ≈ 1.5 *r*_e_), the corrections become large and tend to cancel each other. This characteristic is consistent with changes in the *T*_1_ diagnostic of the CCSD wave function of CO, increasing from 0.007 at *r* = 0.8 Å to 0.084 at *r* = 1.7 Å. At the upper *r* limit, some single and double cluster amplitudes become as large as 0.2, indicating an increasing multireference character of the electronic wave function of CO. This is connected to the well-known tendency of a restricted Hartree–Fock wave function to become a poor CCSD reference function for describing fission of a closed-shell molecule into two open-shell fragments. This is just the case of the CO molecule which in the ground electronic state X^1^Σ^+^ dissociates to give the carbon and oxygen atoms in their ^3^P states.

The potential energy function *V*_BO_(*r*) of CO was further corrected by accounting for the scalar relativistic effects using the exact-2-component (X2C) approach.^28^ The scalar relativistic correction was determined as a difference in the total energy of CO calculated at the CCSD(T)/aug-cc-pV5Z(uncontracted) level of theory using either the X2C or nonrelativistic Hamiltonian. Near the equilibrium configuration of CO, the scalar relativistic correction was determined to be −67.6 m*E*_h_.

Finally, diagonal energy correction *V*_ad_(*r*) was determined for the main isotopologue ^12^C^16^O, as well as for some minor isotopologues. The correction was calculated at the CCSD/aug-cc-pCVQZ level of theory.^29,30^ The calculations were performed using the CFOUR program.^26^ Near the equilibrium configuration of ^12^C^16^O, the diagonal correction was predicted to be 4.14 m*E*_h_. For the minor isotopologues ^13^C^16^O, ^12^C^18^O, and ^13^C^18^O, this correction was calculated to be distinctly different of 4.01, 3.87, and 3.74 m*E*_h_, respectively.

**Figure 2:**
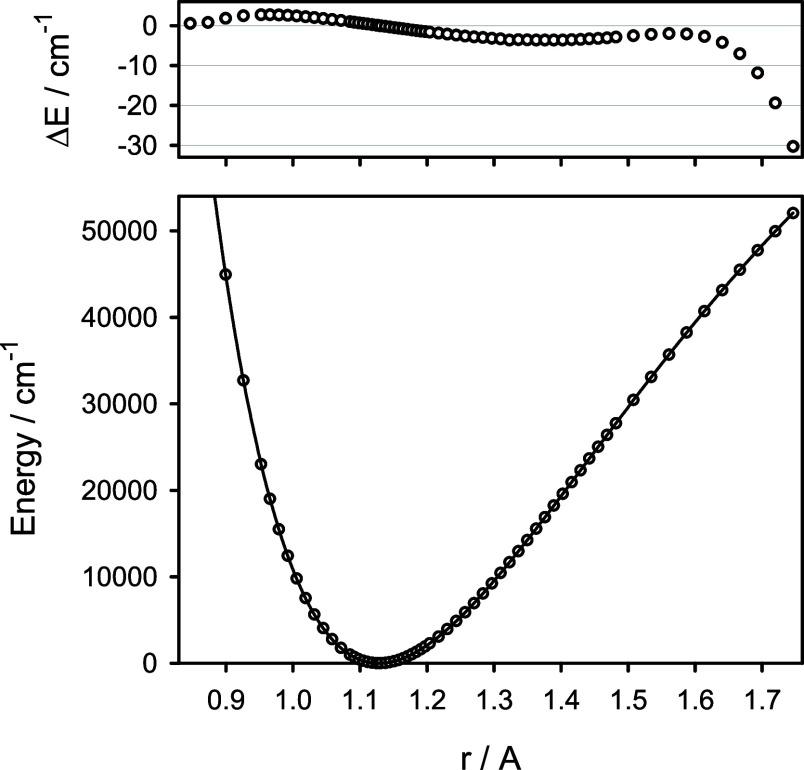
Predicted Born–Oppenheimer potential energy function *V*_BO_(*r*) for the X^1^Σ^+^ state of CO (circles) in comparison to its counterpart derived from experimental data of ref (2). (solid line). The upper panel shows differences between the predicted and experimental values.

**Figure 3:**
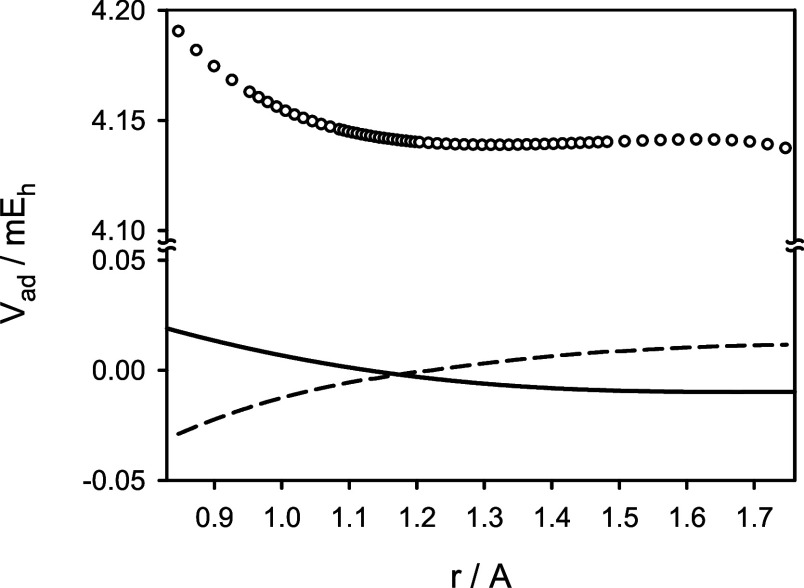
Predicted diagonal energy correction *V*_ad_(*r*) for the X^1^Σ^+^ state of ^12^C^16^O (circles) in comparison to its counterpart derived from experimental data of ref (2) (solid line) and ref (4) (dashed line).

The molecular parameters of ^12^C^16^O predicted with various corrected potential energy functions are given in Table 2. As could be expected, the corrections for higher-order electron correlation affected the parameters the most. The best predicted values are close to those obtained by Coxon and Hajigeorgiou^2^ from the direct-potential-fit analysis of vibration–rotational spectra for seven CO isotopologues. In particular, the calculated BO equilibrium distance *r*_e_ = 1.12825 Å is close to its experimental counterpart, 1.1282295 Å. The adiabatic equilibrium distance *r*_e_ is predicted in this work to be by 0.0000127 Å longer, as compared with the experimentally derived value of 0.0000103 Å.^2^ The predicted BO and adiabatic potential energy functions for the X^1^Σ^+^ state of ^12^C^16^O are given in Table S1 of the Supporting Information. Figure 2 illustrates a comparison of the predicted BO potential energy function with its counterpart derived from the experimental data.^2^ Both functions are essentially identical (on the scale of the figure), with the largest deviations not exceeding 30 cm^–1^ at the upper *r* limit. The diagonal energy corrections *V*_ad_ obtained for various isotopologues of CO are given in Table S2 of the Supporting Information. The function *V*_ad_(*r*) predicted for ^12^C^16^O is shown in Figure 3 and compared with the corresponding mass-dependent terms of the potential energy function derived by Coxon and Hajigeorgiou^2^ and by Meshkov et al.^4^ Note that in the analysis of the experimental data, this term was modeled as a polynomial-type expansion and assumed to vanish at *r* = *∞*. Because the latter is not the case for the diagonal energy corrections *V*_ad_, the theoretically predicted and experimentally derived functions are expected to be shifted in energy. While the predicted function *V*_ad_(*r*) and the corresponding mass-dependent term derived by Coxon and Hajigeorgiou^2^ look alike, the analogous term derived by Meshkov et al.^4^ has just opposite curvature. As a result, the adiabatic equilibrium distance of ^12^C^16^O was found by Meshkov et al.^4^ to be shorter than its BO counterpart. In both experimental studies, the same huge data set was used (seven CO isotopologues, about 21,000 line positions) and reproduced to high spectroscopic accuracy. Even so, the mass-dependent term of the potential energy function of CO was derived in those studies to be qualitatively different. This perfectly exemplifies indeterminacy in the treatment of BO breakdown effects discussed by Watson.^9^ Given the small electric dipole moment of the HD molecule,^31^ the adiabatic effects on the electric dipole moment of CO can be neglected.

**Table 2:** Molecular Parameters^a^ for the X^1^Σ^+^ State of ^12^C^16^O Determined Using Various Potential Energy Functions and Derived from the Experimental Data

	CV^b^	CV + H^c^	CV + H + R^c^	CV + H + R + D^c^	exp.^d^
*r*_e_ (Å)	1.12784	1.12843	1.12825	1.12827	1.1282295
*E* + 113 (hartree)	–0.323070	–0.324136	–0.391719	–0.387576	
ν (cm^–1^)	2150.39	2144.23	2143.33	2143.35	2143.2711
*B*_0_ (cm^–1^)	1.92479	1.92270	1.92329	1.92325	1.9225290
μ (D)	–0.11694	–0.11996	–0.12073		

a See Table 1.

b The potential energy function calculated at the CCSD(T)/aug-cc-pCV8Z level of theory.

c Including additional corrections for the higher-order electron correlation (H), scalar relativistic (R), and adiabatic (D) effects.

d From the direct-potential-fit analysis by Coxon and Hajigeorgiou.^2^

The adiabatic vibrational term values *G*_v_ and the effective rotational constants *B*_v_ predicted for low-lying vibrational levels of the X^1^Σ^+^ state of ^12^C^16^O are given in Table 3. The predicted *G*_v_ values regularly overestimate the corresponding experimental values, the largest deviation being 1.1 cm^–1^ for the highest vibrational level quoted. The predicted *B*_v_ values uniformly overestimate the corresponding experimental values by about 0.0007 cm^–1^, the difference being twice larger than the estimated uncertainty of the theoretical *B*_0_ value.

**Table 3:** Adiabatic Vibrational Term Values (*G*_v_) and Effective Rotational Constants (*B*_v_, All in cm^–1^) for the X^1^Σ^+^ State of ^12^C^16^O

	*G*_v_			*B*_v_		
*v*	exp.^a^	calc.^b^	Δ^c^	exp.	calc.	Δ
0	0.000	0.00	0.00	1.922529	1.92325	0.00072
1	2143.271	2143.26	–0.01	1.905026	1.90574	0.00071
2	4260.062	4260.18	0.12	1.887524	1.88823	0.00071
3	6350.439	6350.74	0.30	1.870024	1.87073	0.00071
4	8414.469	8414.77	0.30	1.852525	1.85323	0.00070
5	10452.222	10452.66	0.43	1.835028	1.83573	0.00070
6	12463.769	12464.31	0.54	1.817533	1.81821	0.00067
7	14449.181	14449.85	0.67	1.800040	1.80076	0.00072
8	16408.535	16409.25	0.71	1.782550	1.78323	0.00068
9	18341.904	18342.82	0.92	1.765062	1.76574	0.00068
10	20249.368	20250.36	0.99	1.747577	1.74828	0.00070
11	22131.005	22132.13	1.12	1.730096	1.73083	0.00073

a The band constants from ref (2), the zero-point energy is 1081.776 cm^–1^.

b The values calculated using the adiabatic potential energy function, the zero-point energy is 1081.80 cm^–1^.

c A difference between the calculated and experimental values.

**Figure 4:**
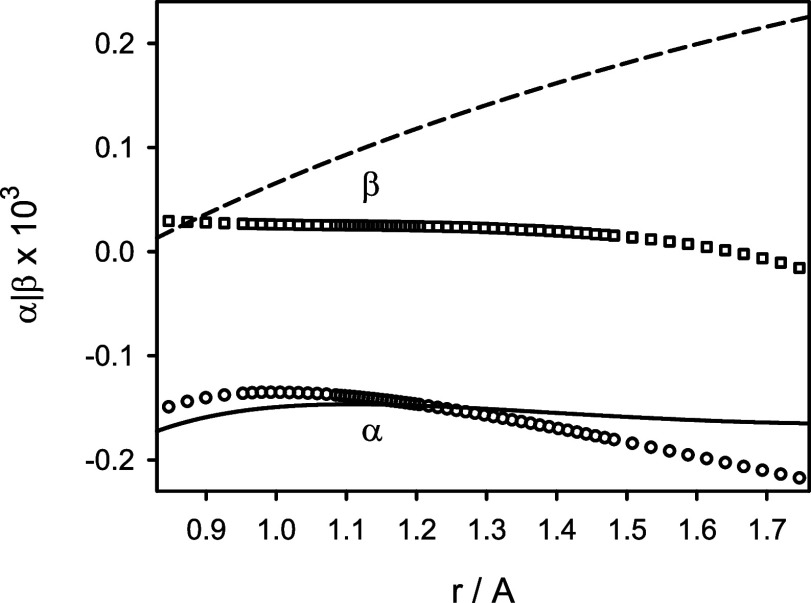
Predicted nonadiabatic parameters α (circles) and β (squares) for the X^1^Σ^+^ state of ^12^C^16^O in comparison to their counterparts derived from experimental data of ref (2) (solid and dashed lines, respectively).

**Figure 5:**
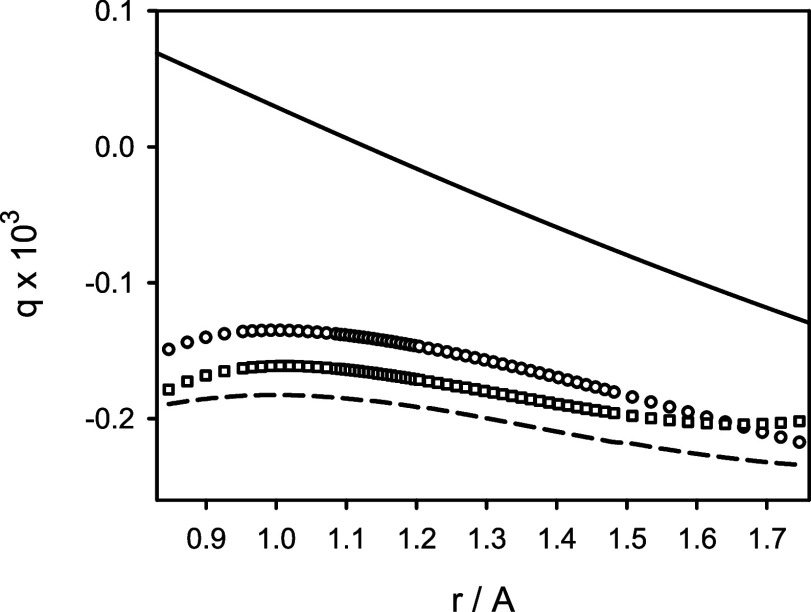
Predicted centrifugal-potential correction functions *g*(*r*) = α(*r*) (circles) and *g*(*r*) = α(*r*) – β(*r*) (squares) for the X^1^Σ^+^ state of ^12^C^16^O in comparison to their counterparts derived from experimental data of ref (2) (solid line) and ref (4) (dashed line).

The effects beyond the adiabatic approximation were investigated by taking into account the nonadiabatic parameters β and α.^11,32−34^ The parameters β and α are related to the electronic contributions to the vibrational and rotational *g*-factors: *g*_v_^el^ and *g*_r_^el^, respectively. These relations are β = (*m*_e_/*m*_p_)*g*_v_^el^ and α = (*m*_e_/*m*_p_)*g*_r_^el^, where *m*_e_/*m*_p_ is the electron–proton mass ratio. The vibrational and rotational *g*-factors for ^12^C^16^O were calculated at various internuclear distances using the complete-active-space self-consistent-field (CASSCF) method with the aug-cc-pV5Z basis set and the full-valence active space consisting of 8 molecular orbitals. The calculations were performed using the DALTON program.^24^ The *g*-factors for the minor isotopologues ^13^C^16^O, ^12^C^18^O, and ^13^C^18^O were also determined, and all are given in Tables S3 and S4 of the Supporting Information. Near the equilibrium configuration of ^12^C^16^O, the parameters β and α were calculated to be about −0.25 × 10^–3^ and −0.41 × 10^–3^, respectively. These parameters are shown in Figure 4 as functions of *r* and compared to those derived by Coxon and Hajigeorgiou.^2^ Because in the analysis of experimental spectra of CO,^2^ the atomic masses of carbon and oxygen were used, the theoretical parameters shown were corrected by adding the (constant) nuclear contribution to the *g*-factors of ^12^C^16^O, (*m*_e_/*m*_p_) *g*^nu^ = 0.27 × 10^–3^. While the predicted and experimentally derived functions α(*r*) look alike, the agreement between the predicted and experimentally derived functions β(*r*) is poor. This is related to the intrinsic indeterminacy of the nonadiabatic parameters included in the effective Hamiltonian *Ĥ*_eff_, in the process of deriving the parameter values from molecular spectra (see Introduction).^9^ For this reason, in the analyses of molecular spectra, an another form of the effective Hamiltonian was often used. In this form, the nonadiabatic terms associated with the kinetic energy operator were incorporated into the rotational part of the potential energy operator as the effective centrifugal-potential correction function [1 + *g*(*r*)].^35^ In general, the nonadiabatic function *g*(*r*) can be expressed in terms of the nonadiabatic functions α(*r*) and β(*r*). In the zeroth-order approximation, *g*(*r*) is identical to α(*r*), whereas in the first-order approximation, *g*(*r*) becomes [α(*r*) – β(*r*)]. A comparison of these two centrifugal-potential correction functions predicted for ^12^C^16^O with the experimentally derived^2,4^ correction functions *g*(*r*) is shown in Figure 5.

The vibrational term values *G*_v_ and the effective rotational constants *B*_v_ predicted taking into account the nonadiabatic effects are given in Table 4. In contrast to the adiabatic approach, the predicted *G*_v_ values regularly underestimate the corresponding experimental values, the largest deviation being 1.4 cm^–1^ for the highest vibrational level quoted. A remarkable change is observed for the rotational constants *B*_v_. The predicted *B*_v_ values underestimate the corresponding experimental values by about 0.00006 cm^–1^, that is, by the order of magnitude better than in the adiabatic approach. The vibrationally averaged internuclear distance ⟨*r*⟩ is calculated for the ground vibrational state of ^12^C^16^O to be 1.132344 Å. For the minor isotopologues ^13^C^16^O, ^12^C^18^O, and ^13^C^18^O, the corresponding ground-state averaged internuclear distances are calculated to be 1.132252, 1.132244, and 1.132150 Å, respectively. The vibrationally averaged rotational and vibrational *g*-factors are predicted to be ⟨*g*_r_⟩ = −0.259 and ⟨*g*_v_⟩ = 0.046, respectively. These values are close to the previous theoretical estimates by Sauer and co-workers^32,34^: *g*_r_ = −0.257 and *g*_v_ = 0.035, as well as to the experimental value of *g*_r_ = −0.26895(5) determined by Meerts et al.^36^ using the molecular-beam electric-resonance technique. For minor isotopologues of CO, Meerts et al.^36^ determined the ^13^C- and ^18^O-isotopic shifts of the rotational *g*-factor to be 0.00945(30) and 0.01270(10), respectively. The corresponding isotopic shifts in the averaged rotational *g*-factors were predicted in this work to be 0.0118 and 0.0119, respectively.

**Table 4:** Nonadiabatic Vibrational Term Values (*G*_v_) and Effective Rotational Constants (*B*_v_, All in cm^–1^) for the X^1^Σ^+^ State of ^12^C^16^O

	*G*_v_			*B*_v_		
*v*	exp.^a^	calc.^b^	Δ^c^	exp.	calc.	Δ
0	0.000	0.00	0.00	1.922529	1.92246	–0.00007
1	2143.271	2143.00	–0.27	1.905026	1.90495	– 0.00008
2	4260.062	4259.66	–0.40	1.887524	1.88745	–0.00007
3	6350.439	6349.97	–0.47	1.870024	1.86996	–0.00006
4	8414.469	8413.75	–0.72	1.852525	1.85246	–0.00006
5	10452.222	10451.40	–0.82	1.835028	1.83497	–0.00006
6	12463.769	12462.82	–0.95	1.817533	1.81746	–0.00008
7	14449.181	14448.14	–1.05	1.800040	1.80002	–0.00002
8	16408.535	16407.31	–1.22	1.782550	1.78250	–0.00005
9	18341.904	18340.68	–1.23	1.765062	1.76502	–0.00005
10	20249.368	20248.01	–1.36	1.747577	1.74756	–0.00001
11	22131.005	22129.57	–1.43	1.730096	1.73012	0.00002

a The band constants from ref (2), the zero-point energy is 1081.776 cm^–1^.

b The values predicted taking into account the nonadiabatic effects, the zero-point energy is 1081.66 cm^–1^.

c A difference between the calculated and experimental values.

The vibrational fundamental wavenumbers ν and the effective ground-state rotational constants *B*_0_ for the X^1^Σ^+^ state of various CO isotopologues are given in Table 5. The values predicted in this work using the adiabatic and nonadiabatic approaches are compared with those determined by Coxon and Hajigeorgiou^2^ in the analysis of vibration–rotational spectra.

**Table 5:** Vibrational Fundamental Wavenumbers (ν) and Effective Ground-State Rotational Constants (*B*_0_, All in cm^–1^) for the X^1^Σ^+^ State of Various CO Isotopologues

	ν			*B*_0_		
isotopologue	exp.^a^	calc.^b^	Δ^c^	exp.	calc.	Δ
Adiabatic						
^12^C^16^O	2143.271	2143.26	–0.01	1.922529	1.92325	0.00072
^13^C^16^O	2096.067	2096.05	–0.02	1.837972	1.83863	0.00066
^12^C^18^O	2092.122	2092.10	–0.02	1.830981	1.83164	0.00066
^13^C^18^O	2043.692	2043.65	–0.04	1.746408	1.74700	0.00059
Nonadiabatic						
^12^C^16^O	2143.271	2143.00	–0.27	1.922529	1.92246	–0.00007
^13^C^16^O	2096.067	2095.80	–0.27	1.837972	1.83790	–0.00007
^12^C^18^O	2092.122	2091.85	–0.27	1.830981	1.83091	–0.00007
^13^C^18^O	2043.692	2043.43	–0.26	1.746408	1.74634	–0.00007

a The experimental spectroscopic constants from ref (2).

b The values predicted using the adiabatic and nonadiabatic approaches.

c A difference between the calculated and experimental values.

**Figure 6:**
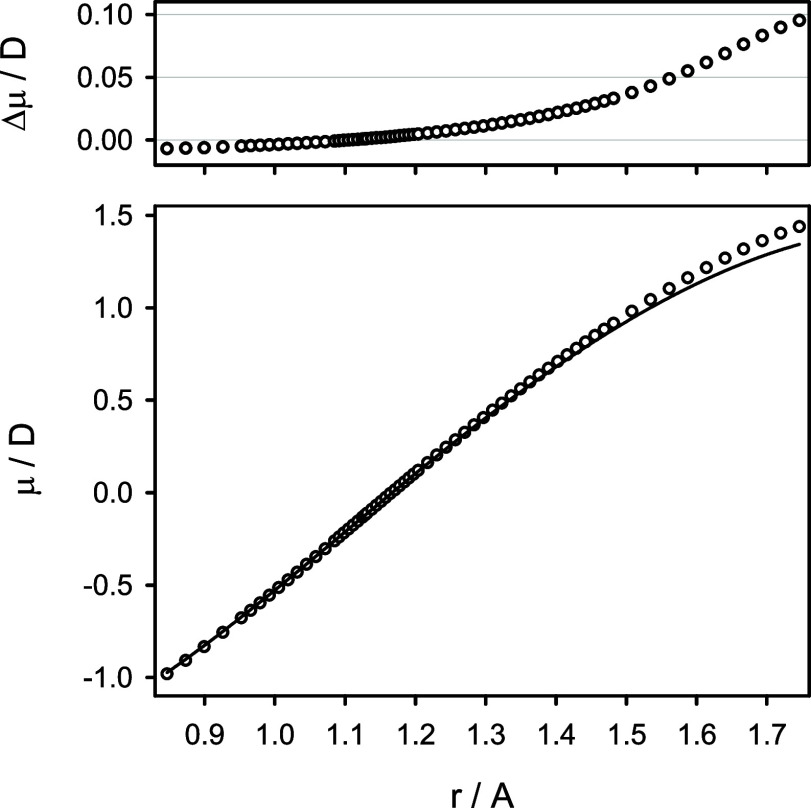
Predicted electric dipole moment function μ(*r*) (circles) for the X^1^Σ^+^ state of CO in comparison to its semiempirical counterpart from ref (5) (solid line). The top panel shows differences between the predicted and experimental values.

The electric dipole moment of CO as a function of the internuclear distance, μ(*r*), was determined first at the CCSD(T)/aug-cc-pCV7Z level of theory, with all electrons included in the correlation treatment. The function μ(*r*) was then corrected for the higher-order electron correlation effects as described above by adding the *E*[CCSDT/aug-cc-pCVQZ] – *E*[CCSD(T)/aug-cc-pCVQZ] and *E*[CCSDTQ/aug-cc-pVTZ] – *E*[CCSDT/aug-cc-pVTZ] energy corrections. Finally, the function μ(*r*) was corrected for the scalar relativistic effects at the CCSD(T)/aug-cc-pV5Z(uncontracted) level of theory. As for the potential energy function of CO, the higher-order electron correlation corrections appeared to be the most important, especially at large internuclear distances (compare Figure 1 above). The predicted electric dipole moment function μ(*r*) of CO in its X^1^Σ^+^ state is given in Table S5 of the Supporting Information. Figure 6 illustrates a comparison of the predicted μ(*r*) function with its semiempirical counterpart derived from the experimental data by Meshkov et al.^5^ Near the equilibrium configuration of CO, both functions differ by about 0.001 D, with the largest deviations not exceeding 0.1 D at the upper *r* limit. The vibrationally averaged electric dipole moment of ^12^C^16^O, ⟨μ⟩_v_, was predicted with the adiabatic vibrational wave functions to be −0.10847, −0.08253, −0.05650, and −0.03038 D for the *v* = 0–3 states, respectively. The ground-state value is close to the experimental value of −0.10980(3) D determined by Muenter.^37^ Meerts et al.^36^ determined this value to be −0.1097(1) D and estimated the vibrational dependence of the averaged electric dipole moment of CO to be ⟨μ⟩_v_ = −0.123 + 0.026(*v* + 1/2) D. Using the theoretical ⟨μ⟩_v_ values quoted above, this dependence is estimated here to be ⟨μ⟩_v_ = −0.12153 + 0.02603 (*v* + 1/2) D.

## Conclusions

The accurate potential energy and electric dipole moment functions of carbon monoxide in the ground electronic state X^1^Σ^+^ were determined in the state-of-the-art ab initio calculations. The mass-dependent terms describing the effects beyond the BO approximation were also determined. The vibration–rotation energy levels of various CO isotopologues were then predicted to a relative accuracy of 10^–4^ or better. The predicted molecular parameters of CO can serve as a good starting point for a further semiempirical analysis of the high-resolution vibration–rotation data.

## Supporting Information Available

Predicted Born–Oppenheimer and adiabatic potential energy functions for ^12^C^16^O in its X^1^Σ^+^ state; predicted diagonal energy correction *V*_ad_(*r*) for ^12^C^16^O, ^13^C^16^O, ^12^C^18^O, and ^13^C^18^O in their X^1^Σ^+^ state; predicted electronic contributions to the rotational *g*-factor for ^12^C^16^O, ^13^C^16^O, ^12^C^18^O, and ^13^C^18^O in their X^1^Σ^+^ state; predicted electronic contributions to the vibrational g-factor for ^12^C^16^O, ^13^C^16^O, ^12^C^18^O, and ^13^C^18^O in their X^1^Σ^+^ state; and the predicted electric dipole moment function of CO in its X^1^Σ^+^ state (PDF)
