# Supplementary material for: Toward Accurate Ab Initio Ground-State Potential Energy and Electric Dipole Moment Functions of Carbon Monoxide
Source: J Chem Theory Comput. 2024 Oct 1;20(20):9041–7. doi: 10.1021/acs.jctc.4c01082 (PMC11500290; doi:10.1021/acs.jctc.4c01082)
Supplement: Supplementary file 1 — ct4c01082_si_001.pdf [file ct4c01082_si_001.pdf]

Toward Accurate Ab Initio Ground-State Potential Energy and Electric Dipole Moment Functions of Carbon Monoxide

Jacek Koput
Department of Chemistry, Adam Mickiewicz University, 61-614 Poznan, Poland

Table S1: The predicted Born-Oppenheimer (CV+H+R) and adiabatic (CV+H+R+D) potential energy functions for $^{12}C^{16}O$ in its X $^1\Sigma^+$ state

r (a.u.)	CV+H+R (a.u.)	CV+H+R+D (a.u.)
1.6000	-113.031607353	-113.027416923
1.6500	-113.117391611	-113.113209688
1.7000	-113.186942319	-113.182767726
1.7500	-113.242736119	-113.238567827
1.7750	-113.266145334	-113.261979849
1.8000	-113.286879629	-113.282716741
1.8250	-113.305153904	-113.300993416
1.8500	-113.321166512	-113.317008241
1.8750	-113.335100607	-113.330944379
1.9000	-113.347125377	-113.342971030
1.9250	-113.357397093	-113.353244475
1.9500	-113.366060153	-113.361909120
1.9750	-113.373247983	-113.369098401
2.0000	-113.379083925	-113.374935669
2.0250	-113.383682001	-113.379534951
2.0500	-113.387147584	-113.383001630
2.0625	-113.388486442	-113.384340996
2.0750	-113.389578143	-113.385433179
2.0875	-113.390433721	-113.386289215
2.1000	-113.391063793	-113.386919722
2.1125	-113.391478548	-113.387334889
2.1250	-113.391687814	-113.387544545
2.1320	-113.391718788	-113.387575728
2.1375	-113.391701043	-113.387558142
2.1500	-113.391527294	-113.387384740
2.1625	-113.391175292	-113.387033066
2.1750	-113.390653445	-113.386511526
2.1875	-113.389969811	-113.385828181
2.2000	-113.389132152	-113.384990793

2.2125	-113.388147917	-113.384006811
2.2250	-113.387024298	-113.382883429
2.2375	-113.385768173	-113.381627524
2.2500	-113.384386165	-113.380245720
2.2625	-113.382884675	-113.378744420
2.2750	-113.381269807	-113.377129726
2.3000	-113.377723256	-113.373583483
2.3250	-113.373790953	-113.369651435
2.3500	-113.369513901	-113.365374590
2.3750	-113.364929931	-113.360790784
2.4000	-113.360073997	-113.355934973
2.4250	-113.354978297	-113.350839359
2.4500	-113.349672538	-113.345533652
2.4750	-113.344184092	-113.340045227
2.5000	-113.338539070	-113.334400199
2.5250	-113.332757858	-113.328618957
2.5500	-113.326864567	-113.322725613
2.5750	-113.320877844	-113.316738818
2.6000	-113.314815653	-113.310676539
2.6250	-113.308694465	-113.304555247
2.6500	-113.302529377	-113.298390043
2.6750	-113.296334210	-113.292194750
2.7000	-113.290121540	-113.285981945
2.7250	-113.283902907	-113.279763170
2.7500	-113.277688828	-113.273548946
2.7750	-113.271488839	-113.267348807
2.8000	-113.265311609	-113.261171426
2.8500	-113.253056285	-113.248915802
2.9000	-113.240977219	-113.236836452
2.9500	-113.229118966	-113.224977947
3.0000	-113.217517338	-113.213376127
3.0500	-113.206201300	-113.202059992
3.1000	-113.195193676	-113.191052423
3.1500	-113.184513205	-113.180372250
3.2000	-113.174174516	-113.170034216
3.2500	-113.164189030	-113.160049871
3.3000	-113.154564846	-113.150427383

Table S2: The predicted diagonal energy correction $V_{ad}(r)$ for $^{12}C^{16}O$, $^{13}C^{16}O$, $^{12}C^{18}O$, and $^{13}C^{18}O$ in their X $^1\Sigma^+$ state

r (a.u.)	$^{12}C^{16}O$	$^{13}C^{16}O$	$^{12}C^{18}O$	$^{13}C^{18}O$
1.6000	0.00419042	0.00405697	0.00391628	0.00378283
1.6500	0.00418192	0.00404883	0.00390820	0.00377511
1.7000	0.00417459	0.00404181	0.00390123	0.00376846
1.7500	0.00416829	0.00403578	0.00389525	0.00376274
1.7750	0.00416548	0.00403319	0.00389269	0.00376019
1.8000	0.00416288	0.00403060	0.00389012	0.00375784
1.8250	0.00416048	0.00402830	0.00388784	0.00375566
1.8500	0.00415827	0.00402617	0.00388574	0.00375365
1.8750	0.00415622	0.00402421	0.00388381	0.00375180
1.9000	0.00415434	0.00402241	0.00388202	0.00375009
1.9250	0.00415261	0.00402075	0.00388039	0.00374853
1.9500	0.00415103	0.00401923	0.00387889	0.00374709
1.9750	0.00414958	0.00401784	0.00387751	0.00374577
2.0000	0.00414825	0.00401656	0.00387626	0.00374457
2.0250	0.00414704	0.00401540	0.00387512	0.00374348
2.0500	0.00414595	0.00401435	0.00387409	0.00374249
2.0625	0.00414544	0.00401386	0.00387361	0.00374203
2.0750	0.00414496	0.00401340	0.00387315	0.00374159
2.0875	0.00414450	0.00401296	0.00387272	0.00374118
2.1000	0.00414407	0.00401254	0.00387231	0.00374078
2.1125	0.00414365	0.00401214	0.00387192	0.00374041
2.1250	0.00414326	0.00401176	0.00387156	0.00374006
2.1320	0.00414306	0.00401156	0.00387138	0.00373987
2.1375	0.00414290	0.00401141	0.00387121	0.00373972
2.1500	0.00414255	0.00401107	0.00387088	0.00373941
2.1625	0.00414222	0.00401076	0.00387058	0.00373911
2.1750	0.00414191	0.00401046	0.00387029	0.00373884
2.1875	0.00414163	0.00401018	0.00387002	0.00373858
2.2000	0.00414135	0.00400992	0.00386977	0.00373833
2.2125	0.00414110	0.00400967	0.00386953	0.00373810
2.2250	0.00414086	0.00400944	0.00386931	0.00373789
2.2375	0.00414064	0.00400923	0.00386910	0.00373769
2.2500	0.00414044	0.00400903	0.00386891	0.00373751
2.2625	0.00414025	0.00400885	0.00386874	0.00373734

2.2750	0.00414008	0.00400868	0.00386858	0.00373718
2.3000	0.00413977	0.00400838	0.00386829	0.00373690
2.3250	0.00413951	0.00400813	0.00386806	0.00373667
2.3500	0.00413931	0.00400792	0.00386787	0.00373649
2.3750	0.00413914	0.00400776	0.00386773	0.00373634
2.4000	0.00413902	0.00400763	0.00386762	0.00373623
2.4250	0.00413893	0.00400755	0.00386755	0.00373616
2.4500	0.00413888	0.00400749	0.00386751	0.00373611
2.4750	0.00413886	0.00400746	0.00386750	0.00373610
2.5000	0.00413887	0.00400746	0.00386751	0.00373611
2.5250	0.00413890	0.00400748	0.00386755	0.00373614
2.5500	0.00413895	0.00400753	0.00386761	0.00373619
2.5750	0.00413902	0.00400759	0.00386769	0.00373626
2.6000	0.00413911	0.00400767	0.00386778	0.00373634
2.6250	0.00413921	0.00400776	0.00386789	0.00373644
2.6500	0.00413933	0.00400786	0.00386801	0.00373654
2.6750	0.00413946	0.00400798	0.00386814	0.00373666
2.7000	0.00413959	0.00400810	0.00386828	0.00373679
2.7250	0.00413973	0.00400823	0.00386842	0.00373692
2.7500	0.00413988	0.00400836	0.00386857	0.00373706
2.7750	0.00414003	0.00400850	0.00386873	0.00373719
2.8000	0.00414018	0.00400863	0.00386888	0.00373734
2.8500	0.00414048	0.00400890	0.00386919	0.00373761
2.9000	0.00414076	0.00400916	0.00386948	0.00373788
2.9500	0.00414101	0.00400938	0.00386974	0.00373811
3.0000	0.00414121	0.00400955	0.00386995	0.00373830
3.0500	0.00414130	0.00400962	0.00387008	0.00373839
3.1000	0.00414125	0.00400955	0.00387006	0.00373835
3.1500	0.00414095	0.00400924	0.00386981	0.00373810
3.2000	0.00414029	0.00400858	0.00386923	0.00373751
3.2500	0.00413915	0.00400745	0.00386820	0.00373649
3.3000	0.00413746	0.00400579	0.00386664	0.00373497

Table S3: The predicted electronic contributions to the rotational g-factor for $^{12}C^{16}O$, $^{13}C^{16}O$, $^{12}C^{18}O$, and $^{13}C^{18}O$ in their X $^1\Sigma^+$ state

r (a.u.)	$^{12}C^{16}O$	$^{13}C^{16}O$	$^{12}C^{18}O$	$^{13}C^{18}O$
1.6000	-0.77771	-0.74265	-0.74445	-0.70716
1.6500	-0.76824	-0.73372	-0.73523	-0.69849
1.7000	-0.76138	-0.72729	-0.72851	-0.69221
1.7500	-0.75666	-0.72291	-0.72383	-0.68786
1.7750	-0.75519	-0.72156	-0.72234	-0.68631
1.8000	-0.75371	-0.72021	-0.72085	-0.68513
1.8250	-0.75281	-0.71940	-0.71990	-0.68428
1.8500	-0.75224	-0.71892	-0.71928	-0.68374
1.8750	-0.75198	-0.71873	-0.71895	-0.68348
1.9000	-0.75199	-0.71879	-0.71888	-0.68347
1.9250	-0.75225	-0.71910	-0.71905	-0.68368
1.9500	-0.75276	-0.71964	-0.71945	-0.68411
1.9750	-0.75347	-0.72037	-0.72006	-0.68474
2.0000	-0.75439	-0.72129	-0.72085	-0.68554
2.0250	-0.75548	-0.72239	-0.72182	-0.68651
2.0500	-0.75674	-0.72364	-0.72295	-0.68764
2.0625	-0.75742	-0.72432	-0.72357	-0.68825
2.0750	-0.75815	-0.72503	-0.72422	-0.68889
2.0875	-0.75891	-0.72579	-0.72491	-0.68957
2.1000	-0.75970	-0.72657	-0.72564	-0.69029
2.1125	-0.76053	-0.72738	-0.72639	-0.69103
2.1250	-0.76139	-0.72822	-0.72717	-0.69179
2.1320	-0.76188	-0.72871	-0.72762	-0.69224
2.1375	-0.76227	-0.72910	-0.72799	-0.69259
2.1500	-0.76319	-0.73000	-0.72883	-0.69342
2.1625	-0.76414	-0.73093	-0.72970	-0.69427
2.1750	-0.76511	-0.73188	-0.73059	-0.69514
2.1875	-0.76611	-0.73285	-0.73151	-0.69604
2.2000	-0.76714	-0.73385	-0.73246	-0.69696
2.2125	-0.76819	-0.73488	-0.73343	-0.69790
2.2250	-0.76926	-0.73593	-0.73442	-0.69887
2.2375	-0.77036	-0.73699	-0.73544	-0.69986
2.2500	-0.77148	-0.73808	-0.73647	-0.70086
2.2625	-0.77262	-0.73919	-0.73753	-0.70189
2.2750	-0.77378	-0.74032	-0.73861	-0.70294

2.3000	-0.77616	-0.74264	-0.74082	-0.70508
2.3250	-0.77861	-0.74502	-0.74310	-0.70730
2.3500	-0.78114	-0.74748	-0.74546	-0.70958
2.3750	-0.78374	-0.74999	-0.74788	-0.71192
2.4000	-0.78640	-0.75257	-0.75036	-0.71432
2.4250	-0.78912	-0.75520	-0.75291	-0.71678
2.4500	-0.79190	-0.75789	-0.75550	-0.71928
2.4750	-0.79473	-0.76063	-0.75816	-0.72184
2.5000	-0.79761	-0.76342	-0.76086	-0.72445
2.5250	-0.80054	-0.76625	-0.76361	-0.72710
2.5500	-0.80352	-0.76912	-0.76640	-0.72979
2.5750	-0.80654	-0.77204	-0.76924	-0.73252
2.6000	-0.80961	-0.77499	-0.77212	-0.73529
2.6250	-0.81271	-0.77799	-0.77503	-0.73810
2.6500	-0.81584	-0.78101	-0.77799	-0.74093
2.6750	-0.81902	-0.78406	-0.78097	-0.74380
2.7000	-0.82222	-0.78715	-0.78399	-0.74670
2.7250	-0.82545	-0.79026	-0.78703	-0.74963
2.7500	-0.82871	-0.79340	-0.79010	-0.75258
2.7750	-0.83199	-0.79656	-0.79320	-0.75555
2.8000	-0.83530	-0.79974	-0.79632	-0.75854
2.8500	-0.84197	-0.80615	-0.80262	-0.76459
2.9000	-0.84870	-0.81262	-0.80898	-0.77069
2.9500	-0.85548	-0.81913	-0.81539	-0.77682
3.0000	-0.86228	-0.82566	-0.82183	-0.78299
3.0500	-0.86909	-0.83220	-0.82828	-0.78917
3.1000	-0.87591	-0.83873	-0.83474	-0.79535
3.1500	-0.88272	-0.84526	-0.84119	-0.80152
3.2000	-0.88952	-0.85178	-0.84764	-0.80769
3.2500	-0.89631	-0.85828	-0.85409	-0.81385
3.3000	-0.90309	-0.86478	-0.86053	-0.82000

Table S4: The predicted electronic contributions to the vibrational g-factor for $^{12}C^{16}O$, $^{13}C^{16}O$, $^{12}C^{18}O$, and $^{13}C^{18}O$ in their X $^1\Sigma^+$ state

r (a.u.)	$^{12}C^{16}O$	$^{13}C^{16}O$	$^{12}C^{18}O$	$^{13}C^{18}O$
1.6000	-0.44980	-0.43352	-0.42585	-0.40740
1.6500	-0.45122	-0.43502	-0.42701	-0.40863
1.7000	-0.45246	-0.43632	-0.42803	-0.40971
1.7500	-0.45358	-0.43749	-0.42895	-0.41068
1.7750	-0.45405	-0.43798	-0.42933	-0.41110
1.8000	-0.45452	-0.43847	-0.42972	-0.41149
1.8250	-0.45493	-0.43890	-0.43006	-0.41185
1.8500	-0.45531	-0.43929	-0.43037	-0.41218
1.8750	-0.45565	-0.43965	-0.43065	-0.41248
1.9000	-0.45596	-0.43997	-0.43090	-0.41275
1.9250	-0.45623	-0.44026	-0.43113	-0.41298
1.9500	-0.45647	-0.44051	-0.43133	-0.41319
1.9750	-0.45669	-0.44074	-0.43150	-0.41338
2.0000	-0.45689	-0.44094	-0.43167	-0.41355
2.0250	-0.45707	-0.44113	-0.43182	-0.41371
2.0500	-0.45723	-0.44130	-0.43196	-0.41385
2.0625	-0.45731	-0.44138	-0.43202	-0.41392
2.0750	-0.45739	-0.44146	-0.43209	-0.41399
2.0875	-0.45747	-0.44154	-0.43216	-0.41406
2.1000	-0.45755	-0.44162	-0.43223	-0.41413
2.1125	-0.45763	-0.44170	-0.43230	-0.41420
2.1250	-0.45771	-0.44178	-0.43237	-0.41427
2.1320	-0.45775	-0.44182	-0.43241	-0.41432
2.1375	-0.45779	-0.44186	-0.43245	-0.41435
2.1500	-0.45791	-0.44197	-0.43256	-0.41445
2.1625	-0.45799	-0.44205	-0.43264	-0.41453
2.1750	-0.45808	-0.44214	-0.43272	-0.41461
2.1875	-0.45818	-0.44223	-0.43281	-0.41470
2.2000	-0.45828	-0.44232	-0.43291	-0.41479
2.2125	-0.45838	-0.44242	-0.43301	-0.41489
2.2250	-0.45849	-0.44253	-0.43312	-0.41499
2.2375	-0.45861	-0.44264	-0.43324	-0.41510
2.2500	-0.45876	-0.44277	-0.43339	-0.41524
2.2625	-0.45890	-0.44290	-0.43353	-0.41536
2.2750	-0.45904	-0.44303	-0.43367	-0.41549

2.3000	-0.45935	-0.44331	-0.43398	-0.41578
2.3250	-0.45970	-0.44363	-0.43434	-0.41610
2.3500	-0.46009	-0.44398	-0.43473	-0.41646
2.3750	-0.46052	-0.44438	-0.43517	-0.41686
2.4000	-0.46098	-0.44479	-0.43564	-0.41729
2.4250	-0.46150	-0.44526	-0.43617	-0.41777
2.4500	-0.46205	-0.44577	-0.43674	-0.41829
2.4750	-0.46266	-0.44632	-0.43736	-0.41885
2.5000	-0.46331	-0.44691	-0.43802	-0.41946
2.5250	-0.46399	-0.44753	-0.43872	-0.42009
2.5500	-0.46477	-0.44824	-0.43951	-0.42081
2.5750	-0.46557	-0.44897	-0.44032	-0.42156
2.6000	-0.46642	-0.44974	-0.44119	-0.42235
2.6250	-0.46733	-0.45057	-0.44211	-0.42319
2.6500	-0.46828	-0.45144	-0.44307	-0.42407
2.6750	-0.46931	-0.45238	-0.44411	-0.42502
2.7000	-0.47040	-0.45338	-0.44522	-0.42603
2.7250	-0.47153	-0.45442	-0.44636	-0.42708
2.7500	-0.47281	-0.45559	-0.44765	-0.42826
2.7750	-0.47413	-0.45680	-0.44897	-0.42948
2.8000	-0.47553	-0.45809	-0.45038	-0.43078
2.8500	-0.47862	-0.46094	-0.45346	-0.43363
2.9000	-0.48212	-0.46419	-0.45695	-0.43686
2.9500	-0.48607	-0.46785	-0.46087	-0.44049
3.0000	-0.49053	-0.47200	-0.46528	-0.44459
3.0500	-0.49571	-0.47683	-0.47038	-0.44935
3.1000	-0.50143	-0.48218	-0.47601	-0.45460
3.1500	-0.50795	-0.48829	-0.48240	-0.46058
3.2000	-0.51529	-0.49517	-0.48957	-0.46730
3.2500	-0.52351	-0.50289	-0.49759	-0.47482
3.3000	-0.53255	-0.51141	-0.50639	-0.48309

Table S5: The predicted electric dipole moment function $\mu(r)$ for CO in its X $^1\Sigma^+$ state

r (a.u.)	μ (a.u.)
1.6000	-0.38556482
1.6500	-0.35723255
1.7000	-0.32785446
1.7500	-0.29754616
1.7750	-0.28208068
1.8000	-0.26642500
1.8250	-0.25059622
1.8500	-0.23460870
1.8750	-0.21847664
1.9000	-0.20221760
1.9250	-0.18584346
1.9500	-0.16937153
1.9750	-0.15281395
2.0000	-0.13618807
2.0250	-0.11950993
2.0500	-0.10278668
2.0625	-0.09441558
2.0750	-0.08603983
2.0875	-0.07766112
2.1000	-0.06928142
2.1125	-0.06090206
2.1250	-0.05252473
2.1320	-0.04783490
2.1375	-0.04415115
2.1500	-0.03578326
2.1625	-0.02742220
2.1750	-0.01907008
2.1875	-0.01072819
2.2000	-0.00239894
2.2125	0.00591867
2.2250	0.01422142
2.2375	0.02250628
2.2500	0.03077388
2.2625	0.03902257
2.2750	0.04725093
2.3000	0.06363924

2.3250	0.07992973
2.3500	0.09611182
2.3750	0.11217532
2.4000	0.12811208
2.4250	0.14391262
2.4500	0.15957048
2.4750	0.17507623
2.5000	0.19042408
2.5250	0.20560725
2.5500	0.22061924
2.5750	0.23545560
2.6000	0.25010487
2.6250	0.26457789
2.6500	0.27885603
2.6750	0.29293999
2.7000	0.30682753
2.7250	0.32051156
2.7500	0.33399140
2.7750	0.34726667
2.8000	0.36033078
2.8500	0.38581845
2.9000	0.41041856
2.9500	0.43410475
3.0000	0.45681616
3.0500	0.47849589
3.1000	0.49899784
3.1500	0.51819218
3.2000	0.53586522
3.2500	0.55180473
3.3000	0.56586050
